# Quality Assessment of Published Systematic Reviews in High Impact Cardiology Journals: Revisiting the Evidence Pyramid

**DOI:** 10.3389/fcvm.2021.671569

**Published:** 2021-06-09

**Authors:** Abdelrahman I. Abushouk, Ismaeel Yunusa, Ahmed O. Elmehrath, Abdelmagid M. Elmatboly, Shady Hany Fayek, Omar M. Abdelfattah, Anas Saad, Toshiaki Isogai, Shashank Shekhar, Ankur Kalra, Grant W. Reed, Rishi Puri, Samir Kapadia

**Affiliations:** ^1^Department of Cardiovascular Medicine, Heart, Vascular & Thoracic Institute, Cleveland Clinic Foundation, Cleveland, OH, United States; ^2^Harvard T.H Chan School of Public Health, Harvard University, Boston, MA, United States; ^3^Center for Outcomes Research and Evaluation, University of South Carolina College of Pharmacy, Columbia, SC, United States; ^4^Faculty of Medicine, Cairo University, Cairo, Egypt; ^5^Faculty of Medicine, Al-Azhar University, Cairo, Egypt; ^6^Department of Internal Medicine, Morristown Medical Center, Morristown, NJ, United States

**Keywords:** cardiology, publication bias, systematic review, quality assessment, critical appraisal

## Abstract

**Objective:** Systematic reviews are increasingly used as sources of evidence in clinical cardiology guidelines. In the present study, we aimed to assess the quality of published systematic reviews in high impact cardiology journals.

**Methods:** We searched PubMed for systematic reviews published between 2010 and 2019 in five general cardiology journals with the highest impact factor (according to Clarivate Analytics 2019). We extracted data on eligibility criteria, methodological characteristics, bias assessments, and sources of funding. Further, we assessed the quality of retrieved reviews using the AMSTAR tool.

**Results:** A total of 352 systematic reviews were assessed. The AMSTAR quality score was low or critically low in 71% (95% CI: 65.7–75.4) of the assessed reviews. Sixty-four reviews (18.2%, 95% CI: 14.5–22.6) registered/published their protocol. Only 221 reviews (62.8%, 95% CI: 57.6–67.7) reported adherence to the EQUATOR checklists, 208 reviews (58.4%, 95% CI: 53.9–64.1) assessed the risk of bias in the included studies, and 177 reviews (52.3%, 95% CI: 45.1–55.5) assessed the risk of publication bias in their primary outcome analysis. The primary outcome was statistically significant in 274 (79.6%, 95% CI: 75.1–83.6) and had statistical heterogeneity in 167 (48.5%, 95% CI: 43.3–53.8) reviews. The use and sources of external funding was not disclosed in 87 reviews (24.7%, 95% CI: 20.5–29.5). Data analysis showed that the existence of publication bias was significantly associated with statistical heterogeneity of the primary outcome and that complex design, larger sample size, and higher AMSTAR quality score were associated with higher citation metrics.

**Conclusion:** Our analysis uncovered widespread gaps in conducting and reporting systematic reviews in cardiology. These findings highlight the importance of rigorous editorial and peer review policies in systematic review publishing, as well as education of the investigators and clinicians on the synthesis and interpretation of evidence.

**Graphical Abstract:**
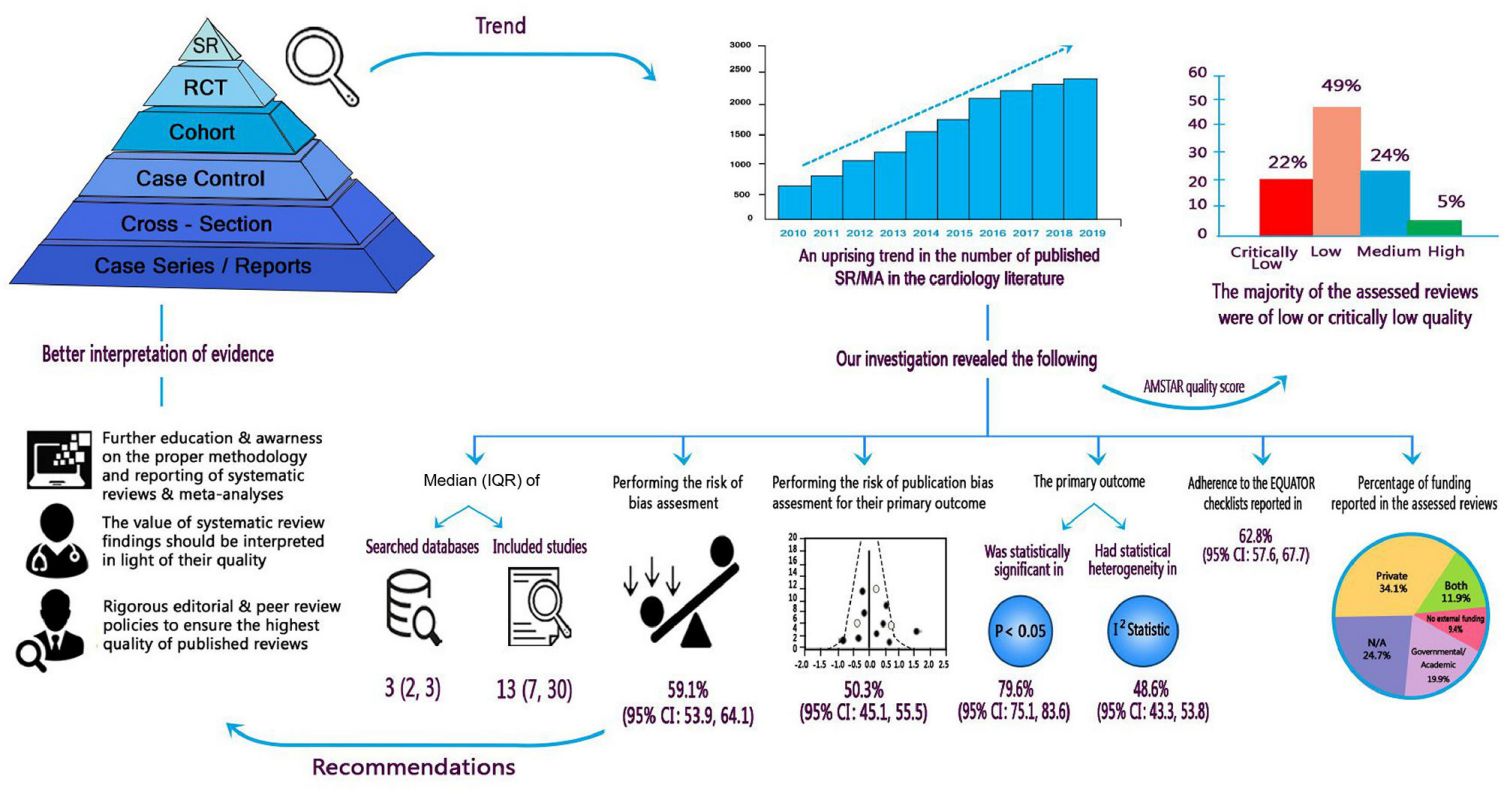
Summary of findings and implications for researchers, clinicians, and stakeholders.

## Introduction

Systematic reviews are conducted to synthesize evidence, identify literature gaps and suggest potential areas for research, in a concerted effort to shape clinical practice guidelines and improve patient care outcomes (1, 2). Given their contribution to informing evidence-based practice, the quality of systematic reviews should not be an acceptable area of compromise as poor quality reviews might contribute to the use of low-efficacy or harmful interventions (3, 4). Several guidelines on the conduct and reporting of systematic reviews have been introduced (5–8). However, adherence to these guidelines has not been optimal. In fact, multiple analyses have shown that the quality of systematic reviews has been declining across different medical specialties (9–19).

Among these specialties, Cardiology has witnessed an exponential growth in the number of published systematic reviews and meta-analyses over the past decade, with >2,400 meta-analyses published in 2019, roughly quadruple the number from 2012 (as per our Medline search). In addition, the clinical practice guidelines in cardiology are increasingly reliant on systematic reviews because they are perceived as the highest level of evidence in the evidence-based pyramid (20–22). Despite increasing publication and utilization, concerns have been raised about the poor quality and low methodological standards of such reviews (11, 23). To our knowledge, only one analysis by Rao et al. surveyed 82 cardiology systematic reviews to determine their overall characteristics without in-depth quality assessment or critical appraisal (23).

Herein, we performed a comprehensive quality assessment of published systematic reviews in high impact, general cardiology/cardiovascular medicine journals. Based on our findings, we provide recommendations for researchers, clinicians, journal editors and peer reviewers, as well as policy makers on the conduct and interpretation of systematic reviews in cardiovascular medicine.

## Materials and Methods

### Search Strategies

We searched PubMed for systematic reviews (with or without meta-analyses) published between 2010 and 2019 in five general cardiology/cardiovascular medicine journals with the highest impact factors according to the 2019 Clarivate Analytics Journal Impact Factor (JIF) list (Circulation [23.6], European Heart Journal [22.7], Journal of the American College of Cardiology [20.6], Circulation Research [14.5], and JAMA Cardiology [12.8]). Although the scope of “Circulation Research” relates mainly to basic cardiovascular research, it publishes systematic reviews of clinical studies on emerging biological and molecular interventions in different cardiovascular diseases. Therefore, it was deemed relevant for this analysis. The detailed search strategies, used in the present analysis, are illustrated in Supplementary Table 1.

A systematic review was defined as per the MeSH database as “A review of primary literature in health and health policy that attempts to identify, appraise, and synthesize all the empirical evidence that meets specified eligibility criteria to answer a given research question,” while a meta-analysis was defined as “Works consisting of studies using a quantitative method of combining the results of independent studies.” Screening for eligible studies was performed by two independent authors (AO and AIA) in two subsequent steps: title and abstract screening followed by full-text screening.

### Data Extraction

The following data were extracted from eligible systematic reviews: Type (direct, individual-patient data [IPD], network meta-analysis [NMA], others), country of origin (classified into single and multinational collaborations), number and type of included studies (randomized trial, observational and diagnostic test accuracy studies), number of searched databases and filters used during the search, whether the reviewers searched protocol registration sites, conference abstracts, and reference lists of retrieved reports, and used risk of bias assessment tools. We further extracted data on the primary outcome, its statistical significance, and the presence of statistical heterogeneity. Further, we checked the sources of funding and classified them if present into governmental/academic and private sources.

In addition, we assessed whether the eligible reviews performed publication bias assessment and the methods used (Egger's test, Begg's test, Funnel plots, correction by trim and fill method, others). For reviews that used any of these tests, we extracted the results of publication bias assessment. For studies that did not perform such assessment, we searched the article for the reason of not performing this analysis.

### Assessment of Systematic Review Quality

We used the “Assessing the Methodological Quality of Systematic Reviews (AMSTAR)” checklist online tool to assess the quality of included systematic reviews (https://amstar.ca/Amstar_Checklist.php). The AMSTAR tool was developed by the Universities of Ottawa, Bristol, Bond, and Toronto to assess the methodological quality of systematic reviews. It consists of 16 questions (majority: yes/no answers) that assess the performance of different steps of the systematic review process as determination of inclusion criteria, screening and data extraction, risk of bias and publication bias assessments, as well as analysis methods. It generates a score for each assessed review as either of high, moderate, low, or critically low quality (5).

### Statistical Analysis

We used R software (version 3.6.3 for Windows) to conduct the statistical analysis. Data were expressed as count (proportion, 95% confidence interval [CI]) or median (interquartile range) for categorical and numerical data, respectively. We used Chi-Square, ordinal logistic, and Poisson regression tests to evaluate the association between methodological characteristics, publication bias assessment, AMSTAR quality scores, and citations (after adjusting for the journal and publication year). We used the Wilson method without continuity correction to calculate 95% confidence intervals for proportions (24). A *p*-value < 0.05 was considered statistically significant.

### Patient and Public Involvement Statement

It was not appropriate or possible to involve patients or the public in the design, or conduct, or reporting, or dissemination plans of our research.

## Results

### Characteristics of the Assessed Reviews

Among 659 retrieved search results, we excluded 278 after title/abstract screening. Eventually, we identified 352 eligible systematic reviews that were published in the target cardiology journals between 2010 and 2019. In systematic reviews that included a meta-analysis, the most frequent type was direct head-to-head comparison, followed by IPD meta-analyses, while only few studies used NMA. Only eight studies performed a qualitative systematic review without meta-analysis. The eligibility criteria in the assessed meta-analyses focused on RCTs (*N* = 164; 46.6%), observational studies (*N* = 104; 17.6%), or both RCTs and observational studies (*N* = 65; 16.8%); Table 1. The majority of eligible reviews (*N* = 193) were the result of a multinational collaboration; other reviews were published most frequently from the United States (*N* = 80), followed by the Netherlands (*N* = 13), Italy (*N* = 12), and Canada (*N* = 12).

**Table 1:** Characteristics and literature search methods of the assessed systematic reviews in the current analysis.

	**Count (%) Total = 352 reviews**
**Meta-analysis Type**	
Direct	275 (78.1)
IPD	59 (16.8)
Network Meta-analysis	8 (2.3)
IPD & NMA	2 (0.6)
SR ONLY	8 (2.3)
**Eligible studies**	
RCTs	164 (46.6)
Observational	104 (29.6)
RCTs and observational	65 (18.5)
DTA	14 (4.0)
Others	5 (1.4)
**Databases Searched**	
Median (IQR)	3 (2,3)^*†*^
**Search Filters**	
Yes	130 (36.9)
No	170 (48.3)
N/A	52 (14.8)
**Type of Search Filter Applied (>1 can be used)**	
English	70/130 (53.9)
Date of publication	36/130 (27.7)
Human	39/130 (30)
Clinical Trial	15/130 (11.5)
**Conference abstracts included**	48 (13.6)
**Manual screening**	219 (62.2)
**No. Of included studies**	
Median (IQR)	13 (7,30)^†^

*Data are presented as count (%) or median (interquartile range)^†^*.

### Evaluation of the Used Methods

Sixty-four reviews (18.2%, 95% CI: 14.5–22.6) registered/published their protocol. The median number of searched databases was 3 (IQR: 2, 3). The frequently searched databases were Medline (*N* = 310; 88.1%), EMBASE (*N* = 196; 55.7%), Cochrane Central Register of Clinical Trials (*N* = 168; 47.7%), and Web of Science (*N* = 49; 13.9%). A full list of the used databases and search engines in the assessed reviews is illustrated in Supplementary Table 2. Among the assessed systematic reviews, 130 (36.9%) used search filters, most frequently limiting search results by language, date of publication, human species, and study type. Manual screening was employed in 219 reviews (62.2%), while 48 (13.6%) reviews added conference abstracts to their eligibility criteria. The median number of included studies was 13 (IQR: 7, 30); Table 1.

Among the retrieved systematic reviews, 208 (58.4%, 95% CI: 53.9–64.1) reported their risk of bias assessment in adequate details using validated tools and 177 (52.3%, 95% CI: 45.1–55.5) examined the risk of publication bias. Among the latter, publication bias was present in 35 reviews. The commonly used methods to assess for publication bias were Funnel plots, Egger's and Begg's tests. Among reviews that did not assess for publication bias, only 21 reviews cited reasons, that were mostly related to the small number of included studies.

The primary outcomes in the assessed reviews were commonly related to mortality and stroke. In 274 (79.6%, 95% CI: 75.1–83.6) reviews, the comparison for the primary outcome was statistically significant and statistical heterogeneity was significant in 167 (48.5%, 95% CI: 43.3–53.8) meta-analyses.

Of the assessed studies, 87 (24.7%, 95% CI: 20.5–29.5) did not report on whether they received funding or not, 33 (9.4%, 95% CI: 6.8–12.9) reported receiving no funding, 70 (19.9%, 95% CI: 16.1–24.4) reviews reported receiving governmental/academic funding, 120 (34.1%, 95% CI: 29.3–39.2) reported private funding from pharmaceutical companies, while 42 (11.9%, 95% CI: 9–15.7) reviews received funding from both sources. The distribution of the number of citations for the assessed reviews was clearly skewed (median 92, IQR: 38, 196); the majority was the publications with smaller number of citations; Table 2.

**Table 2:** Reporting standards of the assessed systematic reviews in the current analysis.

	***N* (Proportion, 95% CI)**
**Protocol Registration**	64 (18.2, 14.5–22.6)
**PRISMA/MOOSE checklist compliance**	221 (62.8, 57.6–67.7)
**Risk of bias assessment**	208 (59.1, 53.9–64.1)
**Publication bias assessment (1ry outcome)**	
Yes	177 (50.3, 45.1–55.5)
**Method of assessment (>1 can be used)**	
Funnel plots	148/177 (83.6, 77.5–88.3)
Egger's test	88/177 (49.7, 42.4–57.0)
Begg's test	50/177 (28.3, 22.1–35.3)
**Assessment Finding**	
Present	35/177 (19.8, 14.6–26.3)
Absent	142/177 (80.2, 73.7–85.4)
**Primary outcome statistically significant**	274/344 (79.6, 75.1–83.6)
**Primary outcome statistical heterogeneity**	167/344 (48.6, 43.3–53.8)
**Primary outcome type (>1 can be used)**	
Mortality	138 (39.2, 34.3–44.4)
Stroke	42 (11.9, 9.0–15.7)
**Funding**	
Governmental/Academic	70 (19.9, 16.1–24.4)
Private	120 (34.1, 29.3–39.2)
Both	42 (11.9, 9.0–15.7)
No external fundingN/A	33 (9.4, 6.8–12.9) 87 (24.7, 20.5–29.5)

*Data are presented as count (proportion, 95% CI)*.

### Quality of the Assessed Reviews

The number of reviews with critically low, low, moderate, and high quality scores were 78 (22.2%, 95% CI: 18–27), 171 (48.6%, 95% CI: 43.2–53.9), 85 (24.1%, 95% CI: 19.6–28.8), and 18 (5.1%, 95% CI: 3.1–8), respectively. This means that the majority of assessed meta-analyses (71%, 95% CI: 65.7–75.4) had low or critically low quality. Only 221 (62.8%, 95% CI: 57.6–67.7) reviews stated clearly that they followed the EQUATOR reporting guidelines (PRISMA, MOOSE, etc.).

### Association Between Methodological Characteristics and Review Quality

We found a significant association between the existence of publication bias and statistical heterogeneity of the primary outcome (OR, 3.93; 95% CI, 1.43–10.85), but not significance of the primary outcome (OR, 1.79; 95% CI, 0.58–5.51). Interestingly, regression analysis showed that meta-analyses with complex design (e.g., IPD), those that included RCTs, included a larger number of studies, had a significant primary outcome, adhered to EQUATOR reporting checklist, or had a higher AMSTAR quality score were associated with a significantly higher citation count (*p* < 0.05) after adjusting for journal and publication year. On the other hand, we found no significant association between AMSTAR quality score and publication journal, registering the review protocol (OR, 1.33; 95% CI, 0.69–2.56), multinational collaboration (OR, 1.09; 95% CI, 0.73–1.61) or receiving private funding (OR, 1.02; 95% CI, 0.03–29.43). Interestingly, the publication year positively predicted AMSTAR quality score (OR, 1.03, 95% CI: 1.02–1.04).

## Discussion

The current analysis of 352 systematic reviews, published in high impact cardiology journals, showed serious gaps in conducting and reporting systematic reviews. These gaps include: (1) protocol registration is often a neglected step of the systematic review process; (2) risk of bias assessment is an integral piece of the systematic review process; however, poor tool selection and reporting of findings influences its credibility; (3) publication bias assessment is often neglected or poorly reported in published systematic reviews; and (4) despite the increasing mandate by publishers, the compliance of published systematic reviews to the EQUATOR network checklists is not yet optimal. Overall, the quality of the assessed systematic reviews was often low or critically low. Although we limited our analysis to the most renowned cardiology journals, we expect published systematic reviews in less reputable journals to have similar gaps and quality scores. However, variations may exist based on the availability of methodological and statistical expertise in the journal editorial and peer review pools.

Several tools have been devised to assess the quality of systematic reviews. The 2009 PRISMA statement was designed to address reporting bias and missing data (6). Most journals now ask authors to be PRISMA-compliant while submitting systematic reviews for publication. However, the adherence is not yet uniform (as per our findings). Moreover, PRISMA does not address the methodological and statistical quality of systematic reviews. Another checklist (AMSTAR) was designed to overcome this caveat (5). In our analysis, almost ¾ of the assessed systematic reviews scored critically low to low quality. Besides cardiology, this phenomenon of declining quality has been observed in other disciplines of clinical medicine (9–19). Berlin et al. linked such occurrence to limited expertise, inaccurate methodology and poor adherence to quality evaluation tools (25). On the positive side, we found that publication year was positively associated with improving review quality score. While the magnitude of this association was small and needs further confirmation, it may reflect increasing expertise of systematic review methods among authors and reviewers.

In addition, the current analysis showed that most published reviews in high-impact cardiology journals do not register or refer to their protocols. Preparing and publishing protocols for systematic reviews can improve the consistency between the team members' decisions, reduce the risk of selective outcome reporting, and eliminate redundant reviews in the literature (26). Ideally, the protocol should be a collaboration between clinicians and methodologists to promote transparency, minimize bias and ensure the reproducibility of the review steps (27). A recommended platform for protocol registration is PROSPERO (Centre for Reviews and Dissemination, York University); however, authors may opt to publish their protocol in a peer-reviewed journal to get expert opinion that might improve the quality of their systematic review (28).

Another aspect, assessed in our analysis, was reporting on publication bias assessment. Publication bias occurs when authors, reviewers, and editors tend to not submit or accept manuscripts for publication according to the direction of the study results. Negative studies are less likely to be published than positive studies and tend to take longer to be published. This can affect the reliability of meta-analyses since those including only positive outcomes are likely to overestimate the true effect of an intervention (29). Thus, assessing publication bias is a fundamental part of the systematic review process. In consistence with prior reports (30–32), our analysis showed that only 52.3% of retrieved systematic reviews in high impact cardiology journals reported on publication bias, further confirming that publication bias reporting is often omitted in systematic review publications.

In a statement from the American Heart Association, Rao and colleagues narratively described the statistical and risk of bias assessment tools in a sample of 82 cardiology meta-analyses published in 2014. The writing group then proceeded to assign methodological standards for conducting systematic reviews that addressed protocol development and dissemination, quality assessment, and choosing appropriate statistical methods (23). Based on our analysis, we stress the importance of some of these recommendations (which are also relevant beyond the field of cardiology):

- Protocol registration improves the methodological quality and reporting standards of systematic reviews. An ideal systematic review protocol requires collaboration between clinicians and methodologists.
- Database search: A minimum of two databases should be searched. The selection of search filters should be justified.
- Additional search: Manual search of the bibliography of relevant studies and searching protocol registries should supplement the electronic database search.
- Risk of bias assessment is a core element of the systematic review process. Further, adopting the GRADE system to assess the certainty of evidence is highly recommended.
- Dealing with heterogeneity: This starts with appreciating clinical and methodological heterogeneity between the included studies before commencing analysis. Using a proper statistical model, as well as exploration of the source of heterogeneity using methods as sensitivity and subgroup analysis or meta-regression are essential.
- Publication bias assessment: The assessment of publication bias is an important step in the systematic review process. If not feasible, the authors should at least mention the reason for not conducting such assessment.
- EQUATOR checklist compliance: Ideally, each manuscript should contain a PRISMA/MOOSE checklist as an appendix. Journal editors should apply stricter editorial rules that enforce compliance with EQUATOR network checklist.
- Funding disclosure: All systematic review funding should be disclosed. Journals should use standardized forms to help authors disclose all potential sources of funding.

### Study Limitations

Although our study, to our knowledge, provides the first comprehensive analysis on the methodological characteristics and gaps of systematic reviews in the cardiology literature, some limitations should be addressed. First, journal selection in the present study was based on the impact factor. While we recognize the limitations of such metric, the selected journals are indeed the most reputable in our field. Second, our analysis focused more on methodological rather than statistical properties of systematic reviews, such as analysis models or effect estimates. Third, the number of reviews with significant publication bias was relatively small to fully analyse the possible contributing factors that can be improved in the systematic review methodology.

## Conclusions

Our analysis uncovered serious gaps with published systematic reviews in the cardiology literature, including issues with protocol registration and conducting and reporting bias assessments. These findings highlight the importance of rigorous editorial and peer review policies in systematic review publishing, as well as education of the investigators and clinicians on the synthesis and interpretation of evidence.

## Data Availability Statement

The original contributions presented in the study are included in the article/Supplementary Material, further inquiries can be directed to the corresponding author.

## Author Contributions

AA: idea conception and study design. OA, AS, IY, AElme, AElma, and SF: data collection and initial drafting. AA and IY: data analysis. TI, SS, GR, RP, AK, and SK: final drafting and revision. All authors contributed to the article and approved the submitted version.

## Conflict of Interest

The authors declare that the research was conducted in the absence of any commercial or financial relationships that could be construed as a potential conflict of interest.
