# Supplementary material for: Quality Assessment of Published Systematic Reviews in High Impact Cardiology Journals: Revisiting the Evidence Pyramid
Source: Front Cardiovasc Med. 2021 Jun 9;8:671569. doi: 10.3389/fcvm.2021.671569 (PMC8220077; doi:10.3389/fcvm.2021.671569)
Supplement: Supplementary file 1 [file Table_1.DOCX]

**Supplementary Table 1:** PubMed search strategies and retrieved results for each journal.

| **Journal** | **Search Strategy** | **Results** |
| --- | --- | --- |
| Journal of the American College of Cardiology | (Meta-analysis[Title/Abstract] OR Meta-analysis[Publication Type] OR "Systematic review"[Title/Abstract]) OR Systematic review[Publication Type] AND "Journal of the American College of Cardiology"[Journal] AND "2010/01/01"[PDat] : "2019/12/31"[PDat] | 205 |
| European Heart Journal | (Meta-analysis[Title/Abstract] OR Meta-analysis[Publication Type] OR "Systematic review"[Title/Abstract] OR Systematic review[Publication Type]) AND "European Heart Journal"[Journal] AND "2010/01/01"[PDat] : "2019/12/31"[PDat] | 173 |
| Circulation | (Meta-analysis[Title/Abstract] OR Meta-analysis[Publication Type] OR "Systematic review"[Title/Abstract] OR Systematic review[Publication Type]) AND "Circulation"[Journal] AND "2010/01/01"[PDat] : "2019/12/31"[PDat] | 212 |
| Circulation Research | (Meta-analysis[Title/Abstract] OR Meta-analysis[Publication Type] OR "Systematic review"[Title/Abstract] OR Systematic review[Publication Type]) AND "Circulation Research"[Journal] AND "2010/01/01"[PDat] : "2019/12/31"[PDat] | 32 |
| JAMA Cardiology | (Meta-analysis[Title/Abstract] OR Meta-analysis[Publication Type] OR "Systematic review"[Title/Abstract] OR Systematic review[Publication Type]) AND "JAMA Cardiology"[Journal] AND "2010/01/01"[PDat] : "2019/12/31"[PDat] | 37 |
