# Supplementary material for: Quality Assessment of Published Systematic Reviews in High Impact Cardiology Journals: Revisiting the Evidence Pyramid
Source: Front Cardiovasc Med. 2021 Jun 9;8:671569. doi: 10.3389/fcvm.2021.671569 (PMC8220077; doi:10.3389/fcvm.2021.671569)
Supplement: Supplementary file 2 [file Table_2.DOCX]

**Supp Table 2:** Used databases and search engines in the assessed systematic reviews.

| **Database** | **Number of reviews using the database (%)** |
| --- | --- |
| Medline | 310 (88.1) |
| EMBASE | 196 (55.7) |
| Cochrane Central | 168 (47.7) |
| Web of Science | 49 (13.9) |
| Clinicaltrials.gov | 38 (10.8) |
| CINAHL | 23 (6.5) |
| Scopus | 22 (6.25) |
| Google Scholar | 13 (3.7) |
| PsycINFO | 11 (3.1) |
| Database of Abstracts of Reviews of Effects | 6 (1.7) |
| BioMed Central | 5 (1.4) |
| Allied and Complementary Medicine Database | 4 (1.1) |
| EBSCO | 3 (0.85) |
| ScienceDirect | 3 (0.85) |
| Current Contents | 3 (0.85) |
| LILACS | 3 (0.85) |
| Science Citation Index | 2 (0.57) |
| National Health Service Economic and Evaluation Database | 2 (0.57) |
| Health Technology Assessment Database | 2 (0.57) |
| Hazardous Substances Databank | 2 (0.57) |
| BIOSIS Previews | 2 (0.57) |
| KoreaMed | 2 (0.57) |
| IndMed | 2 (0.57) |
| Healthcare Management Information Consortium (HMIC) | 1 (0.28) |
| CABI | 1 (0.28) |
| Gray literature sources (SIGLE) | 1 (0.28) |
| NHS Centre for Reviews and Dissemination (CRD) | 1 (0.28) |
| Health technology assessment agencies | 1 (0.28) |
| the National Guideline Clearinghouse | 1 (0.28) |
| The National Library for Health | 1 (0.28) |
| Canadian Medical Association Infobase | 1 (0.28) |
| The Guidelines International Network | 1 (0.28) |
| SciELO | 1 (0.28) |
| POPLINE | 1 (0.28) |
| ProQuest | 1 (0.28) |
| Ingenta | 1 (0.28) |

Data are reported as count (%).
